# mNGS helped diagnose scrub typhus-associated HLH in children: a report of two cases

**DOI:** 10.3389/fpubh.2024.1321123

**Published:** 2024-05-09

**Authors:** Hui Jian, Qiu-xia Yang, Jia-xin Duan, Shu-yu Lai, Guang-lu Che, Jie Teng, Li Chang, Xiao-juan Liu, Li-li Luo, Fang Liu

**Affiliations:** ^1^Department of Laboratory Medicine, West China Second University Hospital, Sichuan University, Chengdu, China; ^2^Key Laboratory of Obstetric and Gynecologic and Pediatric Diseases and Birth Defects of Ministry of Education, Sichuan University, Chengdu, China; ^3^Department of Pediatric Critical Care Medicine, West China Second University Hospital, Sichuan University, Chengdu, China

**Keywords:** scrub typhus, hemophagocytic lymphohistiocytosis, metagenomics next-generation sequencing, *Orientia tsutsugamushi*, sepsis

## Abstract

**Background:**

Scrub typhus, caused by the *Orientia tsutsugamushi (Ot)*, is a widespread vector-borne disease transmitted by chigger mites. Hemophagocytic lymphohistiocytosis (HLH) is considered to be one of the potentially severe complications. The diagnosis of scrub typhus-associated HLH may be overlooked due to the non-specific clinical characteristics and the absence of pathognomonic eschar.

**Case presentation:**

We obtained clinical data from two patients in the South of Sichuan, China. The first case involved a 6-year-old girl who exhibited an unexplained fever and was initially diagnosed with sepsis, HLH, and pulmonary infection. The other patient presented a more severe condition characterized by multiple organ dysfunction and was initially diagnosed with septic shock, sepsis, HLH, acute kidney injury (AKI), and pulmonary infection. At first, a specific examination for scrub typhus was not performed due to the absence of a characteristic eschar. Conventional peripheral blood cultures yielded negative results in both patients, and neither of them responded to routine antibiotics. Fortunately, the causative pathogen *Orientia tsutsugamushi (Ot)* was detected in the plasma samples of both patients using metagenomics next-generation sequencing (mNGS) and further confirmed by polymerase chain reaction. Subsequently, they both were treated with doxycycline and recovered quickly.

**Conclusion:**

The unbiased mNGS provided a clinically actionable diagnosis for an uncommon pathogen-associated infectious disease that had previously evaded conventional diagnostic approaches.

## 1 Introduction

Scrub typhus caused by *Orientia tsutsugamushi (Ot)* is a widespread vector-borne disease transmitted by the chigger mite (1, 2). It is a significant etiology of sudden onset of high fever in several countries within the Asia Pacific region (3). The reported mortality rate for untreated patients is 6%, whereas it is 1.4% for treated patients. Limited evidence suggests a significantly higher mortality rate of up to 70% in the absence of appropriate treatment (4, 5). The clinical signs are characterized by non-specific symptoms, including sudden onset of high fever, pneumonia, headache, and fatigue (6). The pathognomonic eschar is characteristic of scrub typhus. A subset of patients may develop serious complications, such as respiratory distress, acute kidney failure, myocarditis, and encephalopathy (7). These events are more likely to occur in cases that have been misdiagnosed due to the oversight or absence of eschars (8).

Hemophagocytic lymphohistiocytosis (HLH), a potentially severe complication of scrub typhus that predominantly affects children, is a life-threatening disorder that can lead to multiorgan failure and mortality (9). Clinical diagnosis can be challenging due to the overlapping symptoms shared with other infections which may also induce HLH (10). Here, we present two cases of scrub typhus-associated HLH in pediatric patients with sudden onset of fever and multi-systemic involvement. Initially, a specific examination for scrub typhus was not performed due to the absence of a characteristic eschar. However, the successful diagnosis was achieved through the utilization of metagenomic next-generation sequencing (mNGS). Appropriate antibiotics were administered and the patients recovered quickly.

## 2 Case presentation

### 2.1 Case 1

The first patient was a 6-year-old girl who was not known to have suffered from any prior diseases. She lived in Panzhihua, a city located in southwest China, bordering on Yunnan Province-where the scrub typhus is endemic. She experienced a repeated high fever of 39.6°C for a week. She was initially treated with cephalosporin drugs and Motrin suspension (specific drug name and dose are unknown) for a suspected upper respiratory tract infection at a nearby hospital for two days, but the effect was not ideal. Scrub typhus was not initially considered as eschar had not appeared on her body. Her body temperature had risen to 40.6°C. She was transferred to the West China Second University Hospital for further care on August 18, 2023.

**Figure 1:**
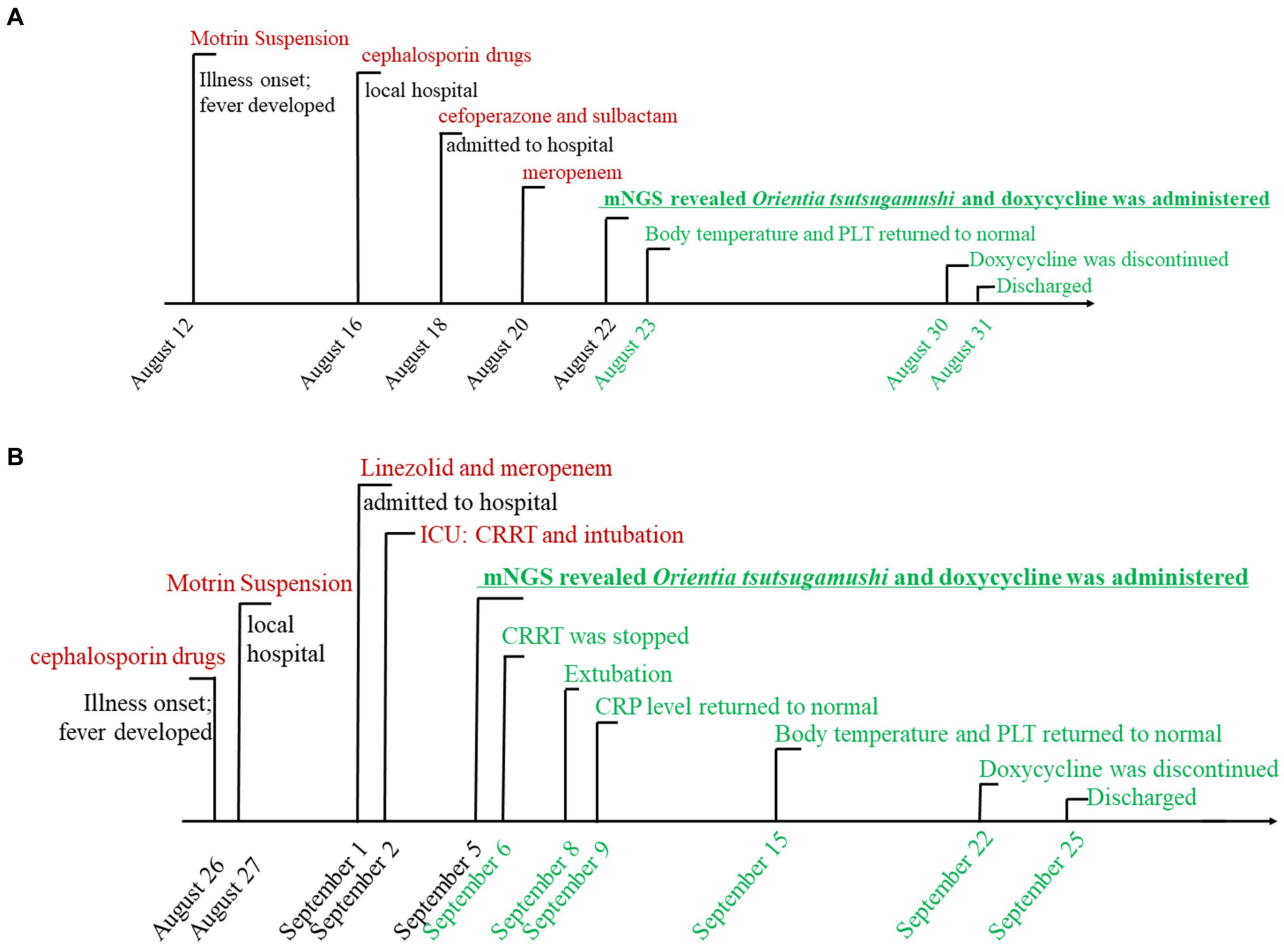
The timeline of case 1 **(A)** and case 2 **(B)** with scrub typhus-associated HLH. Major events during the course of the patient’s illness are described in the line by a different color. Black means deterioration of the disease, red signifies ineffective drug treatment, underlining denotes the turning point, and green represents recovery.

**Figure 2:**
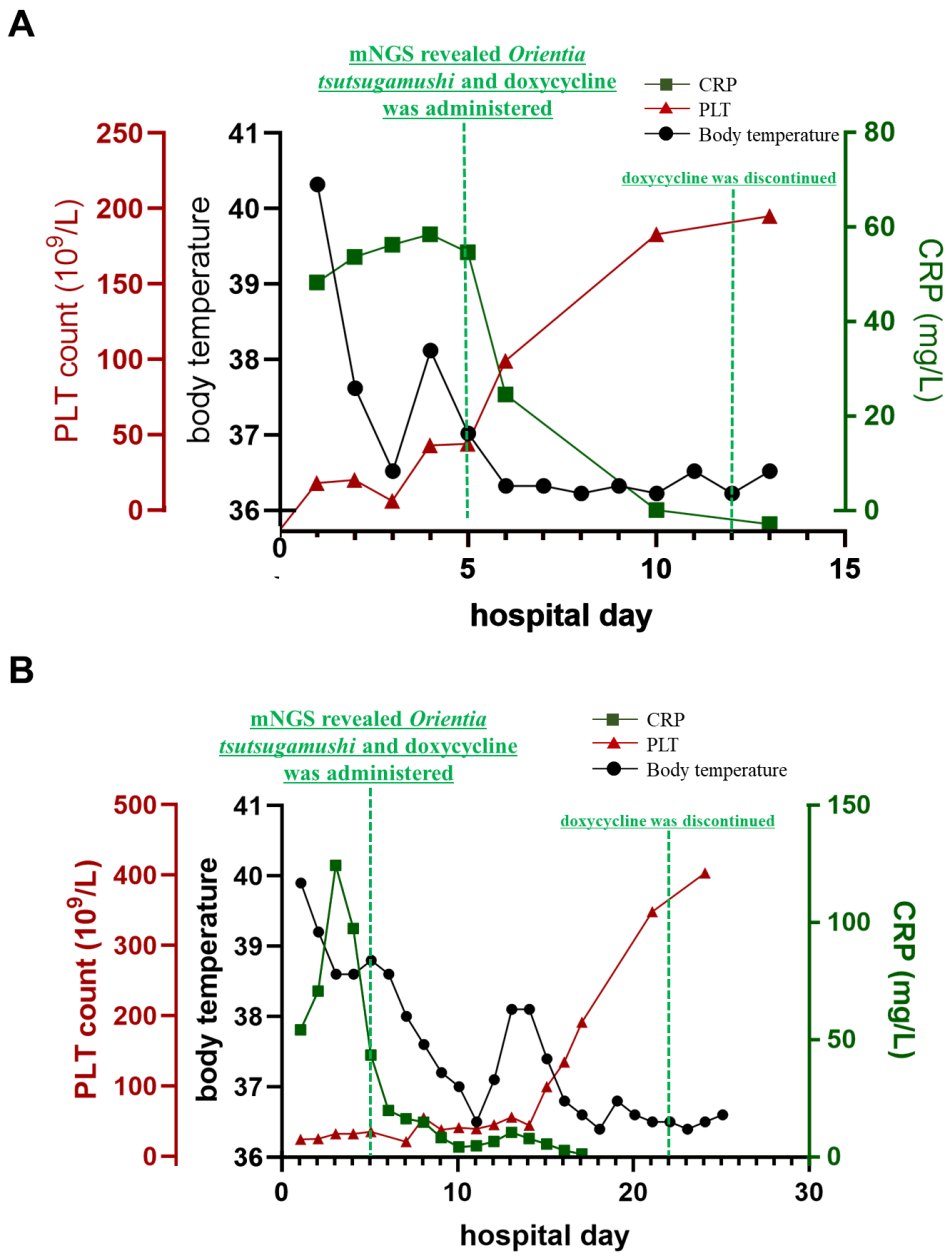
Clinical laboratory examination values during both patients’ hospitalization. C-reactive protein (CRP, green line) and body temperature (black line) were quickly decreased, as well as the serum ferritin line was excluded in this figure. the blood platelet (PLT, red line) was increased obviously own to the treatment of doxycycline.

On admission, routine examination revealed abnormal blood values including elevated CRP levels and lowered hemoglobin and platelet count: hemoglobin was 107 g/L (110–146 g/L), her platelet count was 32 × 10^9^ platelets/L (100–450 × 10^9^/L), and C-reactive protein level was 52.8 (0–8 mg/L). The fibrinolytic system was significantly abnormal: fibrin degradation products were 34.23 (0–5.0 μg/mL) and D-dimer was 10.97 (0–0.5 mg/L, FEU). There were also iron metabolism disturbances, including serum iron (SI 1.08) (7.8–32.3 μmol/L) and serum ferritin (SF 2134.6) (10–291 ng/mL). Other detailed laboratory test results are summarized in Supplementary Table S1. The chest computed tomography (CT) revealed scattered and patchy shadows in both lungs. Bone marrow examination disclosed hemophagocytosis. Hepatosplenomegaly and lymph node enlargement were not observed, and there was no significant ulcer or eschar. The serological, cultural, and polymerase chain reaction (PCR) tests yielded no evidence of other common pathogens, including EBV (Epstein–Barr virus) and TORCH (toxoplasmosis, other, rubella, cytomegalovirus, and herpes). The timeline of case 1 is shown in Figure 1A. The laboratory findings of PLT, CRP, and body temperature during her hospitalization are shown in Figure 2A. Other detailed laboratory test results during hospitalization are summarized in Supplementary Table S1.

Taking into account the patient’s medical background, along with the findings from laboratory and imaging tests, the following initial diagnoses were established: (1) sepsis, (2) HLH, and (3) pulmonary infection. The medication therapy of intravenous cefoperazone and sulbactam (0.9 g intravenous per day) was initiated on the 1st day of admission. However, due to a lack of response to treatment for two consecutive days (with continuous fever exceeding 37.5°C), the antibiotic was changed to meropenem (0.5 g intravenous per day). However, its efficacy remained unsatisfactory.

**Figure 3:**
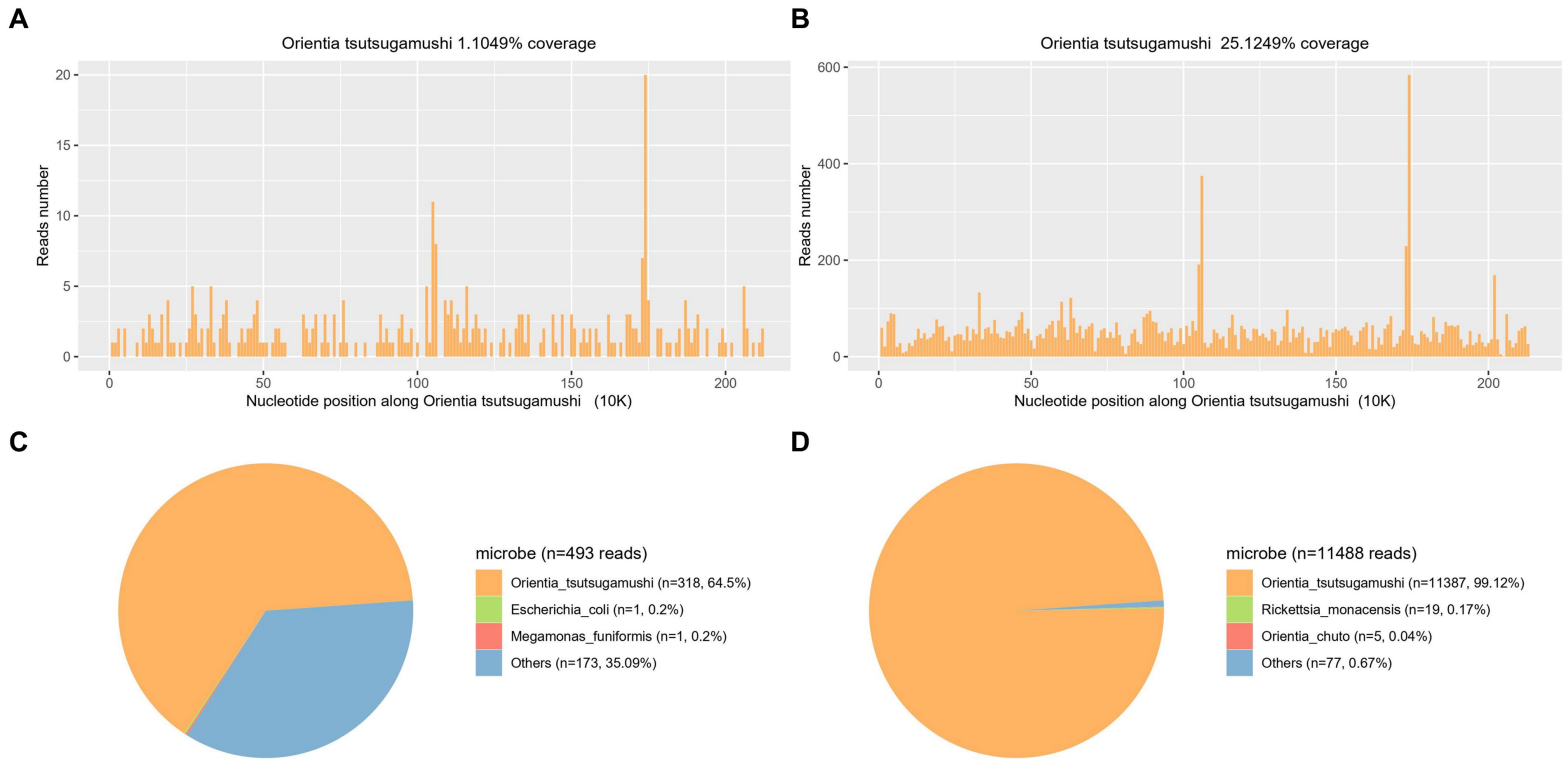
Confirmation of *Ot* in the plasma sample from both patients (**A,C** representing case 1 and **B,D** representing case 2) by mNGS. **A,B** show the reads mapped to *Ot* derived from mNGS data. **C,D** show the distribution of pathogenic microorganism reads in the absence of human, others, and unclassified reads. A total of 318 and 11,387 reads mapped to *Ot* in the reference database which contains about 8,000 pathogen genomes.

In order to make a definitive diagnosis, blood samples were collected on the 4th day of admission (21 August 2023) for mNGS analysis. A detailed protocol is provided in the Supplementary material. Subsequently, on the next day, a total of 318 sequences of *Ot* were detected in the plasma sample (22 August 2023), with a genome coverage and relative abundance of 1.10% and 64.5%, respectively (Figures 3A,C). Consequently, the patient was diagnosed with scrub typhus-associated HLH, and antibiotic treatment was changed to piperacillin-tazobactam combination and doxycycline (20 mg, orally administered twice daily for 8 days). The patient exhibited a rapid recovery of both body temperature and platelet count on the subsequent day (Figure 2A). Other vital signs and the inflammatory indicators gradually returned to within the normal range (Supplementary Table S1). The symptoms gradually resolved, and the administration of doxycycline was discontinued on 30 August 2023, with the subsequent discharge of the patient on the next day. An additional blood sample was collected on the same day as mNGS and was subjected to qPCR validation, for retrospective validation (11). The qPCR protocol is provided in the Supplementary material. The result confirmed the presence of an *Ot* infection with a Ct value of 33.62.

### 2.2 Case 2

A 10-year-old girl was admitted to our hospital with a history of repeated high fever (37.5–39.8°C) and abdominal pain in the right lower quadrant, for one week. This patient lived in Xichang, a city bordering Yunnan Province. Scrub typhus was not initially considered in this case too, as eschar had not appeared on her body. After empirical treatment with cephalosporin drugs and Motrin suspension (specific drug name and dose are unknown), the patient developed dyspnea and multiple organ dysfunction. The patient was then transferred to the intensive care unit (ICU) of our hospital for further treatment.

Physical examination revealed the following vital signs: a temperature of 39.9°C; heart rate of 154/min, respiration rate of 42/min, and a blood pressure of 79/31 mmHg. A blood routine examination showed a platelet count of 25 × 10^9^ /L (100–450 × 10^9^/L). Her fibrinolytic system was significantly abnormal: fibrin degradation products were 51.79 (0–5.0 μg/mL) and D-dimer was 18.87 (0–0.5 mg/L, FEU). Furthermore, the C-reactive protein level was 61.2 (0–8 mg/L) and the serum ferritin level was SF 3163 (10–291 ng/mL). The serum creatinine (Scr) level was 101.79 (17.3–54.6 μmol/L). Other detailed laboratory test results are summarized in Supplementary Table S2. Abdominal computerized tomography revealed hepatosplenomegaly and an enlarged appendix. The chest computed tomography (CT) revealed bilateral pneumonia. Bone marrow examination revealed hemophagocytosis, but malignancy was not observed, and there was no significant ulcer or eschar. The serological examinations yielded negative results for common pathogens, including EBV (Epstein–Barr virus) and TORCH (toxoplasmosis, other, rubella, cytomegalovirus, and herpes). Conventional peripheral blood culture also yielded negative results. The timeline of case 2 is shown in Figure 1B. The laboratory findings of PLT, CRP, and body temperature during her hospitalization are shown in Figure 2B. Other detailed laboratory test results from admission to discharge are summarized in Supplementary Table S2.

Taking into account the patient’s medical background, along with the findings from laboratory and imaging tests, the following initial diagnoses were established: (1) septic shock, (2) sepsis, (3) HLH, (4) acute kidney injury (AKI), and (5) pulmonary infection. The mechanical ventilation and continuous renal replacement therapy (CRRT) were initiated quickly to address multiorgan failure. Linezolid and meropenem (0.5 g intravenous per day) were started as empirical therapy for 4 consecutive days. However, the patient continued to experience persistent fever exceeding 38.5°C.

On day 4 after admission, blood samples were collected and sent for mNGS analysis (4 September 2023). After 24 h, on 5 September 2023, the analysis revealed a total of 11,387 sequences of *Ot*, with a genome coverage and relative abundance of 25.12 and 99.12%, respectively (Figures 3B,D). Consequently, Scrub typhus-associated HLH was diagnosed, and antibiotic treatment was changed to doxycycline (20 mg, orally administered twice daily). The C-reactive protein level exhibited a rapid decline and returned to baseline after 4 days of treatment (9 September 2023). Subsequently, following a 10-day treatment period, the patient’s body temperature and platelet count gradually normalized by 15 September 2023 (Figure 2B). The symptoms gradually resolved while other blood and biochemical indicators exhibited a gradual recovery (Supplementary Table S2). The administration of doxycycline was discontinued on 22 September 2023, and the patient was discharged three days later. After 4 days of doxycycline treatment (9th September), an additional blood sample was collected and subjected to qPCR for retrospective validation (11). The qPCR result validated the finding of mNGS analysis with a Ct value of 33.96.

## 3 Discussion

Diagnosis of scrub typhus may be overlooked unless there is a significant level of suspicion, due to the non-specific clinical features. A typical eschar is a key to diagnosis, however, it was not observed in either of the two cases. The results of culture, serology, and PCR for other epidemic pathogens were all negative. While we had no idea about the causative agent, 318 and 11,387 *Ot* reads were detected, respectively, by mNGS from the plasma samples of both patients. Appropriate antibiotics were administered and the patients recovered quickly.

It has been reported that weather factors are significantly associated with the occurrence and transmission of scrub typhus in China (12). The Southern region of China exhibits a bimodal seasonal pattern, characterized by a large peak in June and a small peak in September (13). Yunnan province stands out as one of the predominant endemic regions in China (14). Both patients in our study came from cities adjacent to Yunnan Province. Clinical presentations included pathognomonic eschar, as well as symptoms such as elevated body temperature, headache, and other general flu-like signs (15). Eschar is a characteristic clinical manifestation and has been reported in varying percentages (ranging from 1 to 97%) of patients depending on geographic regions and studies (16–19). Elevated body temperature is commonly observed in approximately 95–100% of confirmed cases. Neither patient in our study had a specific eschar, and due to the non-specific clinical manifestations during the early stage of the disease prior to admission, the diagnosis was overlooked leading to a rapid progression into sepsis. Relevant knowledge and a comprehensive understanding of the diverse manifestations of this pathogen among clinicians are crucial for reducing morbidity and mortality.

The initial perception of HLH was that it primarily affected infants with mutations in *PRF1, UNC13D,* and *STX11* genes (20). However, further research has shown that this syndrome can also be found in adolescents and adults, leading to the introduction of the “secondary HLH (SHLH)” (21, 22). The occurrence of SHLH is linked to a range of stimuli, such as pathogen infections, malignant lymphoma, and collagen diseases. Previous studies have demonstrated that scrub typhus can also induce hemophagocytosis during the early stage of the disease (10). The possibility of scrub typhus-associated HLH should be considered in patients presenting unidentifiable fever, significantly increased levels of CRP, pancytopenia, and especially those with suspected exposure history. In our research, both patients were initially overlooked until the mNGS revealed the true pathogen.

In our study, both patients demonstrated remarkable resolution with the administration of appropriate antibiotic therapy. The clinical outcome in scrub typhus-associated HLH was found to compare relatively well with that in HLH associated with other secondary causes (10). The documented overall mortality rate of scrub typhus-associated HLH was 6.7% (10). In comparison, the mortality rate in HLH cases linked to EBV has been documented at 14.1% (23), and approximately 50% in tuberculosis (24). Although the prognosis of scrub typhus illness is generally favorable, severe or fatal complications may arise in cases where misdiagnosis occurs due to the oversight or absence of eschars (25). Many reports have emphasized the significance of early diagnosis and timely treatment in facilitating rapid recovery and reducing mortality rates.

Scrub typhus results in multisystem involvement, such as pulmonary, central nervous, and renal systems, as well as the hematological system (10). Typically Acute Respiratory Distress Syndrome (ARDS), which is frequently observed in pediatric cases, carries a high mortality rate (10, 26). The second patient in our study received one week of invasive artificial ventilation. Neurological impairment is observed in approximately 20% of individuals with scrub typhus, impacting both the central and peripheral nervous systems, frequently resulting in more severe clinical outcomes (27). AKI in scrub typhus is another potentially life-threatening complication (28, 29). The second patient’s renal function was recovered after 5 days of CRRT. Early diagnosis and timely treatment are important for the patient’s rapid recovery.

The diagnostic methods of serology, culture, and PCR were commonly employed but exhibited several limitations in their accuracy and reliability (30). The oldest test in current use is the Weil–Felix reaction, which is inexpensive and easy to perform, and results are available overnight; however, it lacks specificity and sensitivity (31). The indirect fluorescent antibody (IFA) test is more sensitive, and results are available in a couple of hours; however, the test is more expensive and requires considerable training (32). The culture of *Ot* poses significant challenges and risks, necessitating execution at special research institutions (33). PCR is primarily employed as a confirmatory test rather than a screening tool due to the presence of numerous pathogenic bacteria in the clinic (34). The mNGS, a high-throughput, fast, and unbiased DNA/RNA detection method, possesses the potential to comprehensively detect a wide spectrum of pathogens in a single test (35). Notably, mNGS exhibits superior sensitivity compared to conventional culture methods, particularly when dealing with special pathogens and novel organisms (36). In this study, on days 4 and 5 of hospitalization, the causative pathogen was identified as *Ot* through mNGS. This result directly contributed to the patient’s remarkable diagnosis and treatment, leading to a favorable outcome.

In summary, *Orientia tsutsugamushi*, in HLH patients with no typical eschar lesions, was detected using mNGS. This research indicates that mNGS has the potential to serve as a valuable approach for identifying unknown agents, especially when specific clinical symptoms are absent. Early diagnosis and prompt initiation of appropriate therapy are important to avoid further deterioration.

## Data availability statement

The datasets presented in this study can be found in online repositories. The names of the repository/repositories and accession number(s) can be found at: https://ngdc.cncb.ac.cn/gsa/, CRA013188.

## Ethics statement

The studies involving humans were approved by Medicine Ethics Committee of West China Second University Hospital, Sichuan University. The studies were conducted in accordance with the local legislation and institutional requirements. Written informed consent for participation was not required from the participants or the participants’ legal guardians/next of kin in accordance with the national legislation and institutional requirements. Written informed consent was obtained from the individual(s) for the publication of any potentially identifiable images or data included in this article.

## Author contributions

HJ: Writing – original draft. Q-xY: Investigation, Writing – review & editing. J-xD: Data curation, Writing – review & editing. S-yL: Formal analysis, Writing – review & editing. G-lC: Supervision, Writing – review & editing. JT: Methodology, Writing – review & editing. LC: Supervision, Writing – review & editing. X-jL: Funding acquisition, Writing – review & editing. L-lL: Project administration, Writing – review & editing. FL: Supervision, Writing – review & editing.
