# Supplementary material for: mNGS helped diagnose scrub typhus-associated HLH in children: a report of two cases
Source: Front Public Health. 2024 May 9;12:1321123. doi: 10.3389/fpubh.2024.1321123 (PMC11111966; doi:10.3389/fpubh.2024.1321123)
Supplement: Supplementary file 1 [file Data_Sheet_1.docx]

**Supplementary Material**

**Metagenomic Next-generation Sequencing and Analysis**

**Methods:**

**Blood Sample Collection**

Using a disposable syringe, take blood from the elbow vein (≥5 mL for adults and ≥2 mL for children) by venipuncture according to the intravenous injection method into a special tube for blood DNA. Immediately mix the blood upside down 8-10 times after collection to prevent clotting and store at 6 ℃-35 ℃ (avoid violent shaking to prevent hemolysis). The samples were transported at 6 ℃-35 ℃ and the plasma was separated within 96 h.

**Nucleic Acid Extraction**

The samples were sealed aseptically and stored at -20 ℃ or transported on dry ice to Hugobiotech Co., Ltd., (Beijing, China) to perform mNGS detection immediately. The DNA was extracted and purified by taking 600 uL of cell-free supernatant sample according to the instructions of QIAamp DNA Micro Kit (QIAGEN, Hilden, Germany). DNA concentration and quality were checked through Qubit 3.0 Fluoremeter (Invitrogen, Q33216) and agarose gel electrophoresis (Major Science, UVC1-1100).

**Library Generation and Sequencing**

DNA library construction was performed according to the Qiagen library construction kit (QIAseq Ultralow Input Library Kit) operating instructions. Library quality control was performed by Qubit 3.0 Fluoremeter (Invitrogen, Q33216) and Agilent 2100 Bioanalyzer (Agilent Technologies, Palo Alto, USA). Qualified DNA libraries with different barcode tags were pooled and then sequenced using the Illumina Nextseq 550 sequencing platform (Illumina, San Diego, USA) and a SE75bp sequencing strategy.

**Bioinformation Pipeline**

After obtaining the sequencing data, high quality data was generated by filtering out connectors, low quality, low complexity and shorter sequences. Next human-derived sequences matching to the human reference database (hg38) were removed by using SNAP software. The remaining data were then aligned to the microbial genome database using Burrow-Wheeler Alignment. This database contains a large collection of microbial genomes from NCBI containing more than 30,000 microorganisms, including 17,748 species of bacteria, 11,058 species of viruses, 1,134 species of fungi, and 308 species of parasites. The accession number of the reference genome of Orientia tsutsugamushi used in this study is NC 009488.1. The microbial composition of the samples was finally determined. The positive criteria for the mNGS result were set as follows:

1. For bacteria other than TB, fungi other than Cryptococcus and parasites: sequencing coverage in the top 10 of all pathogens detected and not detected in the negative control (NTC); or sample/NTC with an RPM (reads per million mapped reads) ratio greater than 10.

(2) For viruses, tuberculosis and cryptococci: at least 1 specific sequence was detected and not detected in the NTC; or the RPM ratio of sample/NTC was greater than 5.

**qPCR protocol used for confirmatory testing**

A standard qPCR was carried out as reported by Ju Jiang et al. (2022)^[1]^ using the primers (O16s-563F, 5′-GCCTGATCCAGCAATG-3′ and O16s-656R 5′-GGCTTTTTCTGTAGGTAC-3′) and the TaqMan probe (O16s-636 P 5′-FAM-TCATTATCAT CCCTACTAAAAGAGCTTTACA-BHQ-1-3′).

DNA was extracted from 200 μL serum. PCR reaction mixture was prepared as follows: primers and probe at 0.3 μM, MgCl_2_ at 6 mM, and annealing/elongation temperature at 58°C; each 20 μl reaction for all the qPCR assays contained 2 μl of template DNA.

Real time PCR was carried out in Applied Biosystems® 7500 Real-Time PCR System. The thermal cycling conditions were: incubation at 50°C for 2 min (to allow for UDG contained within the master mix to function); initial denaturation at 95°C for 2 min; 45 cycles of denaturation at 95°C for 15 s; and annealing/elongation at 58°C for 30 s.

One positive control (recombinant plasmid containing the target fragment of O. tsutsugamushi DNA) and one negative control (nuclease-free water) provided in the kit were included in each run. The detailed protocol mentioned above has been attached in the Supplementary Material.

[1] JIANG J, MARTíNEZ-VALDEBENITO C, WEITZEL T, et al. Development of a New Genus-Specific Quantitative Real-Time PCR Assay for the Diagnosis of Scrub Typhus in South America [J]. Frontiers in medicine, 2022, 9: 831045.
